# Mental disorders during pregnancy and postpartum period

**DOI:** 10.1192/j.eurpsy.2021.1614

**Published:** 2021-08-13

**Authors:** I. Kammoun, R. Kammoun, O. Maatouk, M. Karoui, K. Ben Salah, F. Ellouze

**Affiliations:** Psychiatry G, RAZI UHC, Tunis, Tunisia

**Keywords:** pregnancy, Postpartum, Mental disorders

## Abstract

**Introduction:**

Mental disorders of pregnancy or postpartum correspond to all the psychopathological states linked to the period of the pregnancy-puerperium. They are the subject of prevention and screening and are currently a public health priority

**Objectives:**

Describe the socio-demographic characteristics of the patients who presented mental disorders during pregnancy and/or postpartum. Identify the various risk factors predisposing to these disorders

**Methods:**

We carried out a retrospective descriptive analytical study including patients who presented mental disorders during their pregnancy or postpartum and who were hospitalized during the period from January to October 2020.We collected 20 patients.

**Results:**

The average age was 39.84 years. Mental disorders were present in 73.7% during the postpartum period. The patients had a personality disorder in 47.7%. They were smokers in 57% of cases. Pregnancy was desired in 73.7% with regular follow-up in 84.2%. Pregnancy was complicated by toxemia in 22% of cases and gestational diabetes in 27% of cases. Delivery was by caesarean section in 68.4% with primiparity in 50%. According to the DSM5, the psychic disorder most often found during pregnancy was the characterized depressive disorder 43%, and during the postpartum we found the brief psychotic episode 42.1%. The treatment was in half of the cases association between antidepressants and antipsychotics. Mental disorders were significantly correlated with the presence of stressful life events during pregnancy (p =0.02)

**Conclusions:**

Mental disorders during pregnancy and postpartum are frequent and important to detect. Early diagnosis and adequate care are the two essential elements that should allow these women to fully experience their motherhood

